# Development and Preliminary Psychometric Testing of an Adult Chronic Kidney Disease Self-Management (CKD-SM) Questionnaire

**DOI:** 10.1177/20543581211063981

**Published:** 2021-12-12

**Authors:** Michelle D. Smekal, Maoliosa Donald, Heather Beanlands, Sharon Straus, Gwen Herrington, Blair Waldvogel, Dwight Sparkes, Maria Delgado, Aminu Bello, Brenda R. Hemmelgarn

**Affiliations:** 1 Department of Medicine, University of Calgary, AB, Canada; 2 Department of Community Health Sciences, University of Calgary, AB, Canada; 3 Daphne Cockwell School of Nursing, Ryerson University, Toronto, ON, Canada; 4 Department of Medicine, University of Toronto, ON, Canada; 5 Li Ka Shing Knowledge Institute, St. Michael’s Hospital, Toronto, ON, Canada; 6 Can-SOLVE CKD Network—Patient Partner, Vancouver, BC, Canada; 7 Department of Medicine, Faculty of Medicine & Dentistry, University of Alberta, Edmonton, Canada

**Keywords:** chronic kidney disease, self-management, patient-reported outcomes, questionnaire development, pre-dialysis

## Abstract

**Background:**

Self-management focused interventions to slow chronic kidney disease (CKD) progression are increasingly common. However, valid self-report instruments to evaluate the effectiveness of self-management interventions in CKD are limited.

**Objective:**

We sought to develop and conduct preliminary psychometric testing of a patient-informed questionnaire to assess aspects of CKD self-management for patients with CKD categories G2-G5 (not on kidney replacement therapy [KRT]).

**Design:**

Self-administered electronic questionnaires (multiphase).

**Setting:**

Online.

**Sample:**

Canadian adults with CKD categories G2-G5 (not on KRT)

**Methods:**

The CKD-SM questionnaire was developed and tested in 4 phases. First, we used a content coverage matrix to identify potential questionnaire items based on existing self-efficacy questionnaires, self-management theories, and patient-identified priorities. Second, the draft questionnaire was reviewed by a multidisciplinary expert panel using percent acceptance to finalize the questionnaire. Third, we tested an electronic version of the questionnaire with patients with CKD, evaluating preliminary psychometric properties including internal consistency, face validity, and content validity. Finally, we tested the questionnaire within a CKD self-management intervention study and collected data on internal consistency, test-retest reliability, and pre-post responsiveness.

**Results:**

We identified 22 potential questionnaire items for the first round of expert panel review. Thirteen items were retained in the first round. Eleven additional items were tested in the second review round and all were retained. Of the 24 items retained following expert review of the questionnaire, 21 had greater than 85% acceptance (content validity index [CVI], 0.75-1.00) and 3 items had 75% acceptance (CVI, 0.5). Thirty patients with CKD from across Canada participated in the pilot testing, and 29 patients participated in the CKD self-management intervention study. In the pilot test, several participants requested inclusion of a question that explicitly addressed mental health; consequently, an additional item relating to mental health was included prior to the intervention study (final questionnaire total was 25 items). Internal consistency (Cronbach α) was high for both the pilot (0.921) and intervention study (0.912). Preintervention test-retest reliability, measured with intraclass correlation coefficient, was acceptable (0.732, 95% confidence interval, 0.686-0.771, *P* < .001), and paired pre/postintervention comparison, measured with Wilcoxon sign-rank, demonstrated significant increases in self-management (*P* < .05) despite stable preintervention test-retest responses. Participants were satisfied with the content, wording, and design.

**Limitations:**

The sample sizes were small for each component of the analysis, and the sampling was consecutive/convenience-based.

**Conclusions:**

We used self-management theories, patient-identified self-management needs, expert review, and conducted preliminary psychometric testing to finalize a CKD self-management questionnaire for patients with G2-G5 CKD (not on KRT). The finalized questionnaire assesses aspects of self-management for individuals with CKD and may be particularly helpful as a tool to evaluate self-management interventions among patients with CKD.

## Abrégé

**Contexte:**

Les interventions focalisées sur la prise en charge par le patient qui visent à ralentir la progression de l’insuffisance rénale chronique (IRC) sont de plus en plus courantes. Il existe cependant peu d’outils d’autodéclaration validés pour évaluer l’efficacité de ces interventions en contexte d’IRC.

**Objectifs:**

Nous souhaitions élaborer un questionnaire destiné aux patients pour évaluer les aspects de la prise en charge de la néphropathie chronique par les patients atteints d’IRC de stade G2-G5 (ne suivant aucune thérapie de remplacement rénal [TRR]), puis réaliser des tests psychométriques préliminaires.

**Conception:**

Questionnaires électroniques autoadministrés (multiphases)

**Cadre:**

En ligne

**Échantillon:**

Des Canadiens adultes atteints d’IRC de grade G2-G5 (ne suivant aucune TRR)

**Méthodologie:**

Le questionnaire CKD-SM a été développé et testé en quatre phases : 1) une matrice de couverture de contenu a été utilisée pour déterminer les possibles éléments du questionnaire à partir des questionnaires d’auto-efficacité existants, des théories de prise en charge par le patient et des priorités identifiées par le patient; 2) le questionnaire préliminaire a été examiné par un groupe d’experts multidisciplinaire et finalisé avec le pourcentage d’acceptation; 3) une version électronique du questionnaire a été testée auprès de patients atteints d’IRC, pour en évaluer les propriétés psychométriques préliminaires, notamment la cohérence interne et la validité de la forme et du contenu, et enfin; 4) le questionnaire a été testé dans le cadre d’une étude portant sur les interventions par le patient en IRC et recueillant des données sur la cohérence interne, la fiabilité test-retest et la réactivité avant et après le test.

**Résultats:**

Nous avons défini 22 questions potentielles lors du premier tour de révision par le groupe d’experts. Treize questions ont été retenues au premier tour; onze questions supplémentaires ont été testées lors de la deuxième révision, et toutes ont été adoptées. Parmi les 24 questions retenues après révision du questionnaire par les experts, 21 présentaient une acceptation supérieure à 85 % (ratio de validité du contenu [RVC] : 0,75-1,00), contre 75 % pour les trois autres (RVC 0,5). Trente patients atteints d’IRC de partout au Canada ont participé à l’essai pilote tandis que 29 ont participé à l’étude d’intervention initiées par le patient en IRC. Pendant l’essai pilote, plusieurs participants ont demandé l’inclusion d’une question traitant explicitement de santé mentale; une question supplémentaire relative à la santé mentale a donc été incluse avant l’étude d’intervention (le questionnaire final comportait 25 questions). La cohérence interne (alpha de Cronbach) était élevée tant pour le pilote (0,921) que pour l’étude d’intervention (0,912). La fiabilité du test-retest préintervention, mesurée avec le coefficient de corrélation intra-classe, s’est avérée acceptable (0,732; IC : 0,686-0,771; p=<0,001) et la comparaison préintervention/post-intervention en paire, mesurée avec le test de rang de Wilcoxon, a montré une augmentation significative des interventions initiées par le patient (p<0,05) malgré des réponses stables au test-retest préintervention. Les participants se sont dits satisfaits du contenu, du libellé et de la conception de l’étude.

**Limites:**

Les échantillons pour chaque composante de l’analyse étaient faibles et l’échantillonnage était consécutif/basé sur la commodité.

**Conclusion:**

Nous avons utilisé des théories de prise en charge par le patient, les besoins identifiés par les patients, une revue par des experts et effectué des tests psychométriques préliminaires pour finaliser un questionnaire de prise en charge initiée par le patient de l’IRC (CKD-SM) pour les patients atteints d’IRC de stade G2-G5 (sans TRR). Le questionnaire finalisé évalue les aspects de l’autogestion chez les personnes atteintes d’IRC et peut être particulièrement utile comme outil d’évaluation de ces interventions chez ces patients.

## Introduction

Chronic kidney disease (CKD) affects approximately 10% of the adult population in Canada and leads to significant morbidity, mortality, and health care resource utilization.^1^ Chronic kidney disease is a chronic condition requiring continuous management; the number of people requiring kidney replacement therapy for kidney failure (dialysis or transplant) is steadily increasing, resulting in poor health outcomes for patients and an unsustainable cost to the health care system.^2,3^ In an effort to reduce the risk of kidney failure, CKD management has shifted toward early identification and initiation of proven preventive activities, such as pharmacological and lifestyle management.^4,5^ By intervening early, often before patients experience many symptoms, the risk of developing end-stage kidney disease (ESKD) can be reduced significantly.^6,7^

Patients with CKD who are not yet on kidney replacement therapy (KRT) or preparing to start KRT often have few symptoms, and consequently, they are less inclined to engage in prevention activities, such as dietary modification.^1^ As a result, interventions aiming to promote self-management soon after CKD diagnosis are increasingly common.^8^ Despite these efforts, sustained behavior change can be challenging, and most interventions have not been rigorously evaluated; therefore, it is challenging to determine which interventions may be effective and why.^8,9^ Compounding this is a lack of valid self-report instruments to evaluate self-management interventions targeted toward patients with CKD who are not on KRT. Most available instruments evaluate interventions targeted for “chronic disease” in general or focus on later stages of CKD when patients are nearing or already receiving dialysis and their self-management needs are very different (such as dialysis-related fistula management and closely monitoring serum potassium and phosphorus levels).^10-18^ Moreover, clinicians and content experts developed many of these existing questionnaires with little, or no, input from patients and caregivers, and so the questionnaires may not reflect patients’ self-management needs and priorities. Four related questionnaires, developed to address CKD awareness and/or self-management,^14,15,19,20^ have been used to assess self-management in CKD. However, these questionnaires were developed and validated in China and Taiwan, which introduces some cultural context and translation challenges that may limit “conceptual equivalence” of the questionnaires in a Canadian context.^21^ Moreover, several important self-management domains identified by patients and caregivers in Canada^22^ are not addressed. As a result, there is a need to develop a patient-informed questionnaire that specifically evaluates CKD self-management behavior in a Canadian context; such an instrument would assist in the evaluation of future self-management interventions and educational initiatives targeted to this population. We therefore aimed to develop and test a patient-informed questionnaire to assess CKD-specific self-management behavior in adults with CKD (CKD categories G2-G5 not on KRT).

## Methods

**Figure 1.**
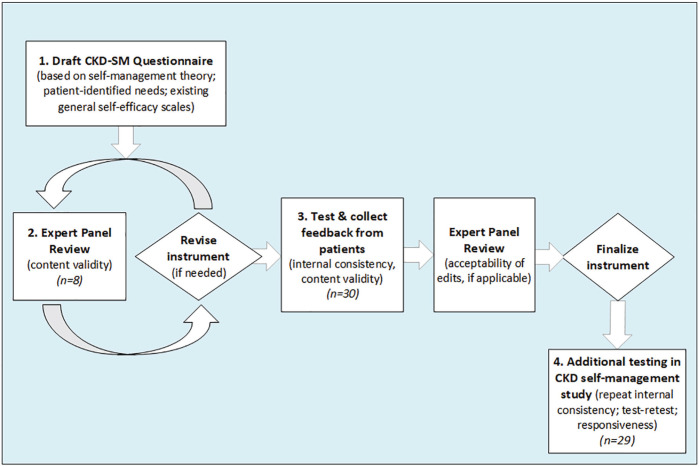
Study design.

The CKD self-management (CKD-SM) questionnaire was developed in 4 phases (Figure 1): (1) item identification; (2) expert panel review; (3) pilot test; and (4) test within a CKD self-management intervention study.

### Phase 1: Item Identification

The purpose of this phase was to review the self-management literature and draft potential questionnaire items for the first round of expert panel review in phase 2.

Self-management is broadly defined as being an active participant in one’s medical care and treatment.^23,24^ Self-management also incorporates aspects of self-efficacy (the belief in one’s ability to take action) and self-care (the ability and confidence to carry out activities that are needed to attain optimal health).^25,26^ We used self-management theoretical frameworks (each incorporating aspects of knowledge, skills, and confidence needed to optimally manage chronic illness; see Box 1), patient-identified self-management priorities identified by Donald et al^22,27^ (understanding CKD, diet, symptoms, medications, physical and mental well-being, finances, travel, work, and education), and reviewed relevant existing self-management questionnaires (Table S1) to guide questionnaire development. Existing self-management/self-efficacy questionnaires were identified using a multi-pronged approach: a MEDLINE and National Institutes of Health National Library of Medicine Health Services and Sciences Research Resources Instrument search (using the search terms self-management, self-care, self-efficacy, kidney) in January 2020.

**Box 1.** Self-Management Theoretical Frameworks Used to Guide Questionnaire Development.

Framework	Description
Self-management tasks^25,28^	Describes 3 critical tasks that a patient must engage in to successfully self-manage: 1. Medical management (such as taking medications) 2. Behavioral management (such as modifying lifestyle) 3. Emotional management (such as managing fear and depression)
Self-management skills^25^	Identifies 5 core skills needed for successful self-management: 1. Problem-solving (seeking solutions) 2. Decision-making (acquiring enough information to respond to changes in their condition) 3. Resource utilization (identifying helpful resources) 4. Patient-provider partnerships (fostering collaborative relationships) 5. Taking action (initiating self-management behaviors)
Self-management processes^29^	Describes 3 key processes needed for self-management: 1. Illness needs (tasks and skills for self-care) 2. Activating resources (identifying and coordinating people involved in care and community services) 3. Living with chronic illness (coping)
Self-management integration^30^	Includes 4 components: 1. Seeking effective self-management strategies (perceived need) 2. Considering the costs and benefits (reflecting on experiences of self-management behaviors) 3. Creating routines and plans of action (actively managing) 4. Negotiating self-management (balancing illness with living a meaningful life)

**Figure 2.**
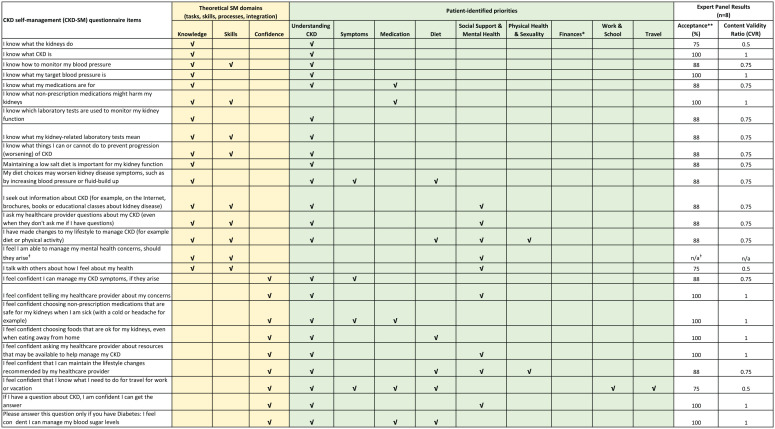
Final questionnaire items and content coverage matrix. *Finances are a patent-identified self-management priority from previous studies; during the expert panel review, questions relating to finances were removed because the panel felt finances were more relevant to later-stage CKD. **Acceptance (%) is the proportion of panel members who indicated whether the item was “relevant” or “highly relevant.” ^ǂ^This question was added after the pilot test (phase 3).

Three research team members (M.D.S., M.D., B.R.H.) iteratively reviewed the potential items using a content coverage matrix (Figure 2) to assess content coverage and representativeness, and to ensure congruence with self-management theoretical framework components and patient-identified priorities. We received preliminary feedback regarding content coverage and representativeness from content experts (3 nephrologists and a health services researcher specializing in CKD self-management who subsequently joined the expert panel), prior to the expert panel review in phase 2 of the study.

Once the potential CKD-SM questionnaire items were identified, we selected a scaling response strategy for the questionnaire. As attitudes and behaviors, such as knowledge and confidence to manage CKD, lie on a continuum,^21^ we selected a continuous direct estimation scaling method to collect participant responses along a 7-point Likert scale ranging from “strongly disagree” to “strongly agree” and “extremely not confident” to “extremely confident.” A 7-point scale was chosen to optimize response reliability while minimizing respondent burden.^21^

### Phase 2: Expert Panel Review and Item Revision

The purpose of this phase was to reach consensus among experts about items and finalize the draft questionnaire for pilot testing with patients.

An expert panel, comprising 8 members (including 4 nephrologists, 2 CKD nurses, 1 patient partner, and 1 CKD self-management researcher), was purposively selected to ensure representation from a variety of clinical roles and prior experience with CKD self-management concepts. The panel was provided with a description of the construct (CKD self-management), the purpose of the tool (to detect whether patients experience a change in self-management behavior following an intervention), and a draft of the CKD-SM questionnaire items. Each round of review was conducted via an electronic questionnaire using Qualtrics software (Qualtrics, Provo, Utah). Panel members were asked to rate each item on a 4-point Likert scale ranging from “not relevant” to “highly relevant” for self-management in early-stage CKD (defined as patients with CKD categories G2-G5 not on KRT or preparing for KRT). If a panel member selected “not relevant” or “somewhat relevant” for an item, they were prompted to record a rationale. An open-ended question was also included for panel members to suggest additional items they felt were an important aspect of CKD self-management. The questionnaire was modified following feedback, including deletion of items scored “not relevant” or “somewhat relevant” by a majority (>50%) of the panel and addition of newly suggested items. The panel independently reviewed the modified questionnaire items iteratively until the content validity ratio (CVR) was ≥0.75 (recommended for panels with 7-9 raters), indicating the majority of reviewers believed the item was essential.^21,31^ Items with a CVR <0.75 were considered for retention if their removal compromised an important content domain (content validity) outlined in the content coverage matrix (Figure 2).

### Phase 3: Pilot Test

The purpose of the pilot test was to evaluate reliability and validity with patients and collect information on understandability, phrasing, and potential self-management content gaps that may lead to identification of additional questionnaire items.

The CKD-SM questionnaire was pilot tested with a convenience sample of Canadian, English-speaking adults with CKD (non-dialysis) via an electronic questionnaire advertised by the Interdisciplinary Chronic Disease Collaboration (ICDC; https://cumming.ucalgary.ca/research/icdc) in a single tweet on Twitter in April 2020. Respondents who self-identified as having been diagnosed with CKD and not on dialysis were eligible to participate. Questionnaires were collected anonymously using Qualtrics survey software (Qualtrics). We sought to include 30 participants in the pilot test; this sample size was chosen based on similar studies suggesting that a sample size of 30 to 50 is adequate for a “pretest” of this nature.^32^ In addition to completing the CKD-SM questionnaire, participants were asked open-ended questions relating to clarity of the questions, comprehension, spelling/word familiarity, ease of response, and whether there were any content/questions missing that they felt were important to self-management in CKD. Questionnaire response reliability was evaluated using the Cronbach α test for internal consistency (to determine whether the items consistently measured the same construct). A Cronbach α value greater than 0.70 was considered acceptable.^33^ Responses to open-ended questions were categorized using a descriptive, conventional content analysis approach.^34^

#### Phase 4: Embed in a CKD-SM Intervention

The purpose of this phase was to pilot test the CKD-SM questionnaire within a self-management intervention study and collect psychometric data relating to reliability, stability, and responsiveness. As the CKD-SM questionnaire was under development, we did not seek to evaluate the self-management intervention with this questionnaire (a separate study to evaluate the feasibility of the self-management intervention was underway and is published elsewhere^35^); rather, the intervention allowed an opportunity to collect additional psychometric data on the instrument itself.

During the self-management intervention, participants had self-directed access to an electronic self-management tool, the *My Kidneys My Health* website (www.mykidneysmyhealth.com), for 8 weeks.^35^ The website was co-designed with patients, researchers, and clinicians with an aim to provide CKD self-management support through education and interactive tools (such as kidney-friendly food lists, nonprescription medication guidance, and depression screening).^36^

The CKD-SM questionnaire was administered twice in the preintervention period, at baseline and 1 week later (test-retest), and postintervention approximately 8 weeks after participants obtained access to the Web-based support tool. The study included a convenience sample of Canadian, English-speaking adults with CKD (non-dialysis) recruited online via Twitter and organizational Web sites (ICDC, Can-SOLVE CKD Network, and the Kidney Foundation of Canada) and through advertisements in 2 nephrology clinics in Alberta from June 2020 to December 2020. Questionnaires were emailed to participants at each time point and completed electronically using Research Electronic Data Capture (RedCap) 10 data capture software (Vanderbilt University Medical Center). Analysis included internal consistency reliability (Cronbach α) and test-retest reliability using a 2-way mixed, absolute effects model (intraclass correlation coefficient [ICC]) to determine whether respondents’ scores were stable between administrations prior to the self-management intervention and pre-post test scores (Wilcoxon sign-rank test). Cronbach α and ICC values greater than 0.7 were considered acceptable.^33,37^ Responsiveness was considered acceptable if preintervention test-retest responses were stable (ICC >0.7) but paired pre-post intervention responses were significantly different (*P* ≤ .05), suggesting a measurable change in self-management following the intervention.

This study was reviewed and approved by the University of Calgary Conjoint Research Ethics Board (CHREB), REB20-0153. Consent was collected and documented for all study participants (implied consent was collected as a component of the anonymous electronic questionnaire for pilot test participants in phase 3, and oral consent was collected and documented for CKD self-management intervention study participants in phase 4).

## Results

### Phases 1 and 2: Item Identification and Expert Panel Review

Following review of existing chronic disease self-efficacy questionnaires and patient-identified priorities, 22 potential questionnaire items were drafted for the first round of expert panel review (Table S2). A panel of 8 content experts (including 4 nephrologists, 2 CKD nurses, 1 patient partner, and 1 CKD self-management researcher) reviewed the draft CKD-SM questionnaire. Two rounds of review were completed; of the 22 items reviewed in the first round, 13 items were retained. In the second round, 11 items were reviewed and, initially, 10 were retained; following review of the content coverage matrix (Figure 2), the item relating to travel and work was retained to preserve content validity overall. Of the 24 items retained following expert panel review of the questionnaire, 21 had >85% acceptance (CVR ≥0.75) and 3 had 75% acceptance (CVR ≥0.50). The 3 items with CVR <0.75 were retained to preserve content validity. Following expert panel review, the finalized questionnaire included content related to knowledge of CKD, skills/confidence to engage in CKD self-management, blood pressure targets, laboratory measurements, nonprescription medications, symptoms, diet, lifestyle factors, communicating needs, and information-seeking behavior.

### Phase 3: Pilot Test

Thirty patients with CKD from across Canada participated in the pilot test from April 2020 to May 2020 (Table 1). Approximately half were men (14/30; 46%) and between 50 and 64 years of age (13/30; 43%). Approximately 40% (12/30) had an estimated glomerular filtration rate (eGFR) of <30 mL/min/1.73 m^2^; the remainder had an eGFR of ≥30 mL/min/1.73 m^2^ or did not know their eGFR. Internal consistency was high (Cronbach α = 0.921). Participants indicated they were satisfied with the content, wording, and design; one participant indicated the questionnaire was “Very quick and easy” (pt 105) and another stated they “thought it was really good. It is progressive, one question leads to the next in terms of relevance” (pt 102) (Table S3). A common comment was the need for a question that more explicitly addressed mental health; for example, one participant responded, “There are no questions about mental health, about family and support systems as CKD affects the whole family” (pt 110) and another asked, “Why is everyone afraid to deal with the mental health issues we face?” (pt 129). Based on this feedback and review with the expert panel, we included an additional item relating to mental health in the final questionnaire: “I feel I am able to manage my mental health concerns, should they arise” (Figure 2) prior to the intervention study in phase 4 (bringing the total number of questions in the final questionnaire to 25 items); see Figure S1 for a hard copy version of the final questionnaire.

**Table 1.** Participant Demographics.

	Pilot (n = 30)		CKD study (n = 29)		Total (n = 59)	
	n	%	n	%	n	%
Age						
<25	1	3.3	0	0	1	1.7
25-49	9	30.0	6	20.7	15	25.4
50-64	13	43.3	11	37.9	24	40.7
65-74	4	13.3	5	17.2	9	15.3
75+	0	0.0	7	24.1	7	11.9
Did not answer	3	10.0	0	0	3	5.1
Sex						
Male	14	46.7	15	51.7	29	49.2
Female	12	40.0	14	48.3	26	44.1
Did not answer	4	13.3	0	0	4	6.8
Employment						
Full-time	7	23.3	11	39.3	18	30.5
Part-time	4	13.3	0	0	4	6.8
Retired	11	36.7	13	46.4	24	40.7
Not employed	2	6.7	1	3.6	3	5.1
Other	3	10.0	3	10.7	6	10.2
did not answer	3	10.0	1	3.6	4	6.8
Marital status						
Married	16	53.3	19	65.5	35	59.3
Single	6	20.0	6	20.7	12	20.3
Divorced	2	6.7	2	6.9	4	6.8
Other	3	10.0	2	6.9	5	8.5
Did not answer	3	10.0	0	0	3	5.1
Education						
≤Grade 12	7	23.3	4	13.8	11	18.6
College, university, trades	17	56.7	17	58.6	34	57.6
Graduate school	3	10.0	8	27.6	11	18.6
Did not answer	3	10.0	0	0	3	5.1
Urban/Rural						
Urban >500 000	15	50.0	17	58.6	32	54.2
Rural <500 000	12	40.0	12	41.4	24	40.7
Did not answer	3	10.0	0	0	3	5.1
Province						
Alberta	9	30.0	22	75.9	31	52.5
British Columbia	3	10.0	3	10.3	6	10.2
Saskatchewan	1	3.3	0	0.0	1	1.7
Ontario	10	33.3	2	6.9	12	20.3
Quebec	1	3.3	0	0.0	1	1.7
New Brunswick	2	6.7	1	3.4	3	5.1
Newfoundland	1	3.3	0	0.0	1	1.7
Nova Scotia	0	0.0	1	3.4	1	1.7
Did not answer	3	10.0	0	0	3	5.1
Ethnicity (not collected in pilot)						
White/Caucasian	—	—	24	82.8	24	82.8
Visible Minority	—	—	3	10.3	3	10.3
Did not answer	—	—	2	6.9	2	6.9
Cause(s) of CKD						
Diabetes	1	3.3	8	27.6	9	15.3
Hypertension	3	10.0	7	24.1	10	16.9
Glomerulonephritis/Inflammatory condition	6	20.0	1	3.4	7	11.9
Obstruction	1	3.3	2	6.9	3	5.1
PKD	4	13.3	3	10.3	7	11.9
Unknown	6	20.0	10	34.5	16	27.1
Other	8	26.7	7	24.1	15	25.4
Did not answer	3	10.0	0	0	3	5.1
Years since diagnosis						
≤5	7	23.3	18	62.1	25	42.4
6-10	4	13.3	3	10.3	7	11.9
≥11	16	53.3	7	24.1	23	39.0
Unknown	0	0.0	1	3.3	1	1.7
Did not answer	3	10.0	0	0	3	5.1
Estimated glomerular filtration rate (mL/min^2^)						
>60	1	3.3	3	10.3	4	6.8
30-60	7	23.3	7	24.1	14	23.7
15-29	3	10.0	5	17.2	8	13.6
<15	9	30.0	6	20.7	15	25.4
Unknown	7	23.3	8	27.6	15	25.4
Did not answer	3	10.0	0	0	3	5.1

*Note.* CKD = chronic kidney disease; PKD = polycystic kidney disease.

### Phase 4: Test Embedded in a CKD-SM Intervention Study

Twenty-nine patients from across Canada participated in the CKD self-management intervention study from June 2020 to February 2021 (Table 1). Approximately half were men (15/29; 52%) and between 50 and 64 years of age (11/29; 38%). Approximately 40% (11/29) had an eGFR of <30 mL/min/1.73 m^2^ and 60% (18/29) had an eGFR of ≥30 mL/min/1.73 m^2^ or did not know their eGFR. Internal consistency reliability (Cronbach α) was 0.912 (Table 2). Test-retest reliability, measured approximately 1 week apart (preintervention) using ICC, was 0.732 (95% confidence interval, 0.686-0.77) (*P* < .001). Twenty-two paired pre/postintervention responses, measured approximately 2 months apart using Wilcoxon sign-rank test, demonstrated significant improvements (*P* < .05) in self-management for 8 items (Table 3) despite stable preintervention test-retest scores; no significant change was observed for the remaining 17 items. Seven participants lost to follow-up did not provide a reason for nonresponse. Two reminder emails were sent to these participants and then they were marked as lost to follow-up.

**Table 2.** Internal Consistency Reliability of the CKD Self-Management (CKD-SM) Questionnaire at 2 Independent Administrations: Pilot Study and Test Within a CKD Self-Management Study.

Item		Pilot (n = 30)				CKD study baseline (n = 29)			
		No. of items missing^a^	Mean (SD)	Cronbach α if item deleted	Cronbach α (overall)	No. of items missing^a^	Mean (SD)	Cronbach α if item deleted	Cronbach α (overall)
Knowledge and Skills	I know what the kidneys do	0	6.27 (0.91)	0.914	**0.921**	0	6.00 (0.71)	0.905	**0.912**
	I know what CKD is	0	6.40 (0.77)	0.912		0	5.45 (1.40)	0.9	
	I know how to monitor my blood pressure	2	6.29 (1.05)	0.912		0	5.93 (1.28)	0.906	
	I know what my target blood pressure is	2	6.25 (1.18)	0.909		0	6.00 (1.34)	0.903	
	I know what my medications are for	0	6.33 (0.84)	0.91		0	5.93 (1.41)	0.907	
	I know what nonprescription medications might harm my kidneys	0	5.90 (1.09)	0.912		0	5.00 (1.54)	0.9	
	I know which laboratory tests are used to monitor my kidney function	0	6.27 (0.91)	0.909		0	5.38 (1.35)	0.901	
	I know what my kidney-related laboratory tests mean	0	5.67 (1.42)	0.907		0	4.86 (1.43)	0.9	
	I know what things I can or cannot do to prevent progression (worsening) of CKD	1	5.62 (1.35)	0.906		0	5.03 (1.35)	0.898	
	Maintaining a low salt diet is important for my kidney function	0	6.50 (0.82)	0.917		0	6.45 (0.74)	0.903	
	My diet choices may worsen kidney disease symptoms, such as by increasing blood pressure or fluid build-up	0	6.53 (0.68)	0.911		0	5.90 (1.32)	0.915	
	I seek out information about CKD (eg, on the Internet, brochures, books, or educational classes about kidney disease)	1	6.00 (1.34)	0.917		0	5.07 (1.44)	0.903	
	I ask my health care provider questions about my CKD (even when they do not ask me whether I have questions)	0	6.13 (0.90)	0.913		0	5.45 (1.53)	0.901	
	I have made changes to my lifestyle to manage CKD (eg, diet or physical activity)	0	6.13 (1.14)	0.911		0	5.66 (1.05)	0.904	
	I feel I am able to manage my mental health concerns, should they arise^b^	n/a	n/a	n/a		0	5.79 (1.18)	0.905	
	I talk with others about how I feel about my health	0	5.53 (1.61)	0.914		0	5.21 (0.90)	0.905	
Confidence	I feel confident I can manage my CKD symptoms, if they arise	0	5.27 (1.62)	0.911		0	4.93 (1.03)	0.902	
	I feel confident telling my health care provider about my concerns	0	6.00 (1.34)	0.91		0	6.21 (0.62)	0.902	
	I feel confident choosing nonprescription medications that are safe for my kidneys when I am sick (eg, with a cold or headache)	0	5.97 (0.96)	0.913		0	4.72 (1.67)	0.9	
	I feel confident choosing foods that are OK for my kidneys, even when eating away from home	0	5.77 (1.17)	0.909		0	5.14 (1.18)	0.9	
	I feel confident asking my health care provider about resources that may be available to help manage my CKD	0	6.03 (1.03)	0.911		0	5.66 (1.08)	0.904	
	I feel confident that I can maintain the lifestyle changes recommended by my health care provider	0	5.93 (0.79)	0.913		0	5.31 (1.14)	0.905	
	I feel confident that I know what I need to do for travel for work or vacation	2	6.04 (0.88)	0.911		0	5.17 (1.14)	0.901	
	If I have a question about CKD, I am confident I can get the answer	0	5.97 (1.19)	0.912		0	5.55 (1.12)	0.906	
	Please answer this question only if you have Diabetes: I feel confident I can manage my blood sugar levels^c^	27	6.00 (1.00)	n/a		19	5.60 (0.52)	n/a	

*Note.* CKD = chronic kidney disease.

a Any administrations with a missing field were omitted from the analysis.

b This item was added after the pilot test, based on recommendations from participants.

c Excluded from statistical analysis due to low response (3 responses in pilot, 10 responses in CKD study).

**Table 3.** Pre-Post Difference in CKD-SM Questionnaire Scores at Baseline (Preintervention) and Approximately 8 Weeks Postintervention (n = 22 Matched Pairs).

CKD-SM questionnaire question			Preintervention mean (SD)	Postintervention mean (SD)	Pre-post change *P* value^a^
Knowledge and Skills	1	I know what the kidneys do	6.05 (0.58)	6.23 (0.69)	.344
	2	I know what CKD is	5.64 (0.90)	6.23 (0.75)	**.014**
	3	I know how to monitor my blood pressure	5.95 (1.36)	6.45 (0.67)	.062
	4	I know what my target blood pressure is	6.09 (1.34)	6.41 (0.67)	.359
	5	I know what my medications are for	6.14 (1.25)	6.32 (0.65)	1
	6	I know what nonprescription medications might harm my kidneys	4.95 (1.62)	5.95 (0.49)	**.024**
	7	I know which laboratory tests are used to monitor my kidney function	5.64 (0.95)	6.05 (0.90)	.063
	8	I know what my kidney-related laboratory tests mean	5.14 (1.13)	5.82 (0.91)	**.002**
	9	I know what things I can or cannot do to prevent progression (worsening) of CKD	5.18 (1.22)	5.76 (0.94)	**.012**
	10	Maintaining a low salt diet is important for my kidney function	6.45 (0.74)	6.68 (0.57)	.188
	11	My diet choices may worsen kidney disease symptoms, such as by increasing blood pressure or fluid build-up	5.95 (1.36)	6.45 (0.60)	.148
	12	I seek out information about CKD (eg, on the Internet, brochures, books, or educational classes about kidney disease)	5.09 (1.38)	5.73 (1.39)	**.016**
	13	I ask my health care provider questions about my CKD (even when they do not ask me whether I have questions)	5.73 (1.42)	6.18 (1.01)	.781
	14	I have made changes to my lifestyle to manage CKD (for example diet or physical activity)	5.59 (1.14)	6.14 (0.89)	**.014**
	15	I feel I am able to manage my mental health concerns, should they arise	5.86 (1.17)	5.55 (1.06)	.121
	16	I talk with others about how I feel about my health	5.14 (0.94)	5.14 (0.99)	1
Confidence	17	I feel confident I can manage my CKD symptoms, if they arise	5.00 (1.07)	5.32 (1.09)	.183
	18	I feel confident telling my health care provider about my concerns	6.32 (0.57)	6.18 (0.66)	.453
	19	I feel confident choosing nonprescription medications that are safe for my kidneys when I am sick (eg, with a cold or headache)	4.59 (1.68)	5.32 (0.95)	.127
	20	I feel confident choosing foods that are OK for my kidneys, even when eating away from home	5.23 (0.97)	5.82 (0.66)	**.009**
	21	I feel confident asking my health care provider about resources that may be available to help manage my CKD	5.64 (1.09)	6.00 (0.69)	.148
	22	I feel confident that I can maintain the lifestyle changes recommended by my healthcare provider	5.41 (1.01)	5.95 (0.49)	**.006**
	23	I feel confident that I know what I need to do for travel for work or vacation	5.27 (0.98)	5.50 (1.01)	.172
	24	If I have a question about CKD, I am confident I can get the answer	5.45 (1.26)	5.86 (0.94)	.148
	25	Please answer this question only if you have Diabetes: I feel confident I can manage my blood sugar levels	5.63 (0.52)	5.88 (0.83)	.625

*Note.* CKD-SM = CKD self-management; CKD = chronic kidney disease;

a We used nonparametric methods (the Wilcoxon sign-rank test for matched pairs) to analyze the Likert scale data. Significant results (*p*<0.05) are highlighted with bold text.

## Discussion

We developed a CKD-SM questionnaire for adults with CKD categories G2-G5 not on KRT and conducted preliminary psychometric testing (including validity, reliability, stability, and responsiveness). The CKD-SM questionnaire was developed through expert consensus, pilot tested with patients, and administered in a pre-post CKD self-management intervention study. Overall, the results suggest the CKD-SM questionnaire performed reliably across multiple administrations, indicating that the CKD-SM questions consistently measured dimensions of the self-management construct. The questionnaire also appeared to be relatively stable across repeated administrations (in the absence of self-management intervention), but was also responsive to potential changes in perceived self-management ability following participation in an electronic self-management intervention. While this study did not evaluate the self-management intervention directly, preliminary results from a feasibility study conducted concurrently with this questionnaire development study suggest the intervention supported patients to manage CKD, particularly with respect to finding and understanding information about CKD, kidney-friendly foods, and choosing nonprescription medications.^35^ The CKD self-management questionnaire items that demonstrated improvement following this intervention reflect improvement in these specific domains, suggesting that the questionnaire was sensitive to the improvements noted by participants in the feasibility study of the self-management intervention.

Studies suggest that effective self-management may slow CKD progression^8,9^; however, there are few measures available to evaluate patient self-management in CKD, particularly for individuals experiencing early stages of CKD, making it challenging to identify effective self-management interventions targeted at this population.^8,9^ Existing measures tend to be nonspecific, evaluating chronic disease broadly, or include questions relating to symptoms and therapies that are common only in later-stage CKD, such as dialysis-related fatigue, fistula management, and monitoring blood phosphorus levels.^10-18^ We identified 3 existing questionnaires that were relevant to CKD self-management for patients not on KRT.^14,15,19^ These questionnaires served as excellent references for item identification for the first iteration of our CKD-SM questionnaire. Although the questionnaires addressed many of the content areas used to develop the CKD-SM questionnaire, there were some gaps across the questionnaires primarily relating to patient-identified self-management domains (such as confidence choosing nonprescription medications, traveling, and managing mental/emotional health) and lack of “conceptual equivalence” for a Canadian context (these questionnaires were developed and validated in China and Taiwan, and some of the translated questions, such as “Do you know how to evaluate your curative effect”^15^ may be unclear to this audience). Our study builds on these earlier questionnaires by attempting to address these gaps and providing a comprehensive self-management questionnaire for patients with CKD who are not on KRT in a Canadian context.

The CKD-SM questionnaire was developed by integrating self-management behavior theories (relating to tasks, skills, processes, and integration^25,28-30^) and by consulting with a panel of clinicians and patients to ensure the questionnaire’s content aligns with what is currently known about patients’ self-management needs in early-stage CKD (ie, understanding CKD, diet, symptoms, medications, physical and mental well-being, finances, travel, work, and education)^22,27^ As a result, the CKD-SM questionnaire provides a patient-informed measure that incorporates aspects of the physical, emotional, and social needs of patients, in addition to the more typically considered clinical aspects related to CKD self-management. As reported by others,^8,38,39^ clinical teams are increasingly acknowledging the importance of incorporating the needs of the “whole person” in self-management interventions. The CKD-SM questionnaire content is a reflection of these broader self-management concepts, including questions relating to social and emotional support, for example, in addition to the more typical clinical outcomes used to assess self-management, such as monitoring blood pressure and laboratory tests.

### Limitations

There are limitations that should be considered when interpreting the results of this study. First, although the questionnaire development was guided by patient priorities and finalized through expert consensus, the sample sizes were small for each component of the analysis and the sampling overall was consecutive/convenience-based. With that in mind, the consistently high Cronbach α results across multiple administrations provide assurance that the internal consistency of the questionnaire is acceptable. Second, our literature review of existing self-management questionnaires was not systematic. Finally, while the study included patients from across Canada, participants had a range of CKD severity (none of the participants were on KRT, however). We attempted to mitigate this in the pilot study by asking patients to reflect on their needs and experiences when they were first diagnosed. As the questionnaire is intended to assess CKD self-management in patients not on KRT, psychometric studies encompassing larger samples sizes and from multiple contexts are needed.

## Conclusion

The CKD-SM questionnaire performed well in preliminary psychometric testing and has the potential to contribute to our understanding of self-management among those with CKD. The questionnaire could be particularly helpful in clinical settings as a baseline measure of perceived ability to manage aspects of CKD that have been identified as important for successful CKD self-management, to monitor and proactively address gaps in knowledge, skills, and confidence to manage CKD, and/or to evaluate self-management interventions. Additional psychometric testing in larger studies will strengthen our understanding of the effectiveness of the questionnaire for measuring CKD self-management; the questionnaire will be further evaluated in self-management intervention studies conducted by our team in the future.

## Supplemental Material

sj-pdf-1-cjk-10.1177_20543581211063981 – Supplemental material for Development and Preliminary Psychometric Testing of an Adult Chronic Kidney Disease Self-Management (CKD-SM) Questionnaire Click here for additional data file. Supplemental material, sj-pdf-1-cjk-10.1177_20543581211063981 for Development and Preliminary Psychometric Testing of an Adult Chronic Kidney Disease Self-Management (CKD-SM) Questionnaire by Michelle D. Smekal, Maoliosa Donald, Heather Beanlands, Sharon Straus, Gwen Herrington, Blair Waldvogel, Dwight Sparkes, Maria Delgado, Aminu Bello and Brenda R. Hemmelgarn in Canadian Journal of Kidney Health and Disease

sj-pdf-2-cjk-10.1177_20543581211063981 – Supplemental material for Development and Preliminary Psychometric Testing of an Adult Chronic Kidney Disease Self-Management (CKD-SM) Questionnaire Click here for additional data file. Supplemental material, sj-pdf-2-cjk-10.1177_20543581211063981 for Development and Preliminary Psychometric Testing of an Adult Chronic Kidney Disease Self-Management (CKD-SM) Questionnaire by Michelle D. Smekal, Maoliosa Donald, Heather Beanlands, Sharon Straus, Gwen Herrington, Blair Waldvogel, Dwight Sparkes, Maria Delgado, Aminu Bello and Brenda R. Hemmelgarn in Canadian Journal of Kidney Health and Disease

sj-pdf-3-cjk-10.1177_20543581211063981 – Supplemental material for Development and Preliminary Psychometric Testing of an Adult Chronic Kidney Disease Self-Management (CKD-SM) Questionnaire Click here for additional data file. Supplemental material, sj-pdf-3-cjk-10.1177_20543581211063981 for Development and Preliminary Psychometric Testing of an Adult Chronic Kidney Disease Self-Management (CKD-SM) Questionnaire by Michelle D. Smekal, Maoliosa Donald, Heather Beanlands, Sharon Straus, Gwen Herrington, Blair Waldvogel, Dwight Sparkes, Maria Delgado, Aminu Bello and Brenda R. Hemmelgarn in Canadian Journal of Kidney Health and Disease

sj-xlsx-4-cjk-10.1177_20543581211063981 – Supplemental material for Development and Preliminary Psychometric Testing of an Adult Chronic Kidney Disease Self-Management (CKD-SM) Questionnaire Click here for additional data file. Supplemental material, sj-xlsx-4-cjk-10.1177_20543581211063981 for Development and Preliminary Psychometric Testing of an Adult Chronic Kidney Disease Self-Management (CKD-SM) Questionnaire by Michelle D. Smekal, Maoliosa Donald, Heather Beanlands, Sharon Straus, Gwen Herrington, Blair Waldvogel, Dwight Sparkes, Maria Delgado, Aminu Bello and Brenda R. Hemmelgarn in Canadian Journal of Kidney Health and Disease
