# Supplementary material for: Development and Preliminary Psychometric Testing of an Adult Chronic Kidney Disease Self-Management (CKD-SM) Questionnaire
Source: Can J Kidney Health Dis. 2021 Dec 12;8:20543581211063981. doi: 10.1177/20543581211063981 (PMC8671825; doi:10.1177/20543581211063981)
Supplement: sj-pdf-2-cjk-10.1177_20543581211063981 – Supplemental material for Development and Preliminary Psychometric Testing of an Adult Chronic Kidney Disease Self-Management (CKD-SM) Questionnaire [file sj-pdf-2-cjk-10.1177_20543581211063981.pdf]

Table S1. CKD-related self-management survey instruments

Instrument	CKD specific?	# of items	Self-Management Domains
Chronic Disease Self-Efficacy Scale (1)	No	32	Exercise, obtaining information/help, communicating with physician, managing disease (general), Doing chores, social/recreational activities, managing symptoms (general), managing shortness of breath, control/manage depression.
CKD Patient Awareness Questionnaire (2)	Yes	18	Symptoms, kidney function, prognosis, blood pressure management, knowing what medications are used for and which medicines are bad for kidneys, kidney-friendly diet, lab tests, how to collect urine, clinics outreach activities, how to contact healthcare team
CKD Self-Management Instrument (3) and CKD Self-Efficacy Instrument (4)	Yes	29 & 25	CKD skills, disease knowledge, communication with healthcare professionals, problem-solving, self-care and autonomy.
CKD self-care scale (5)	Yes	17	Medication, diet control, exercise, smoking behavior
EQ5D (6)	No	5	Mobility, self-care, pain/discomfort, anxiety/depression
IPOS-Renal (Patient Version) (7)	Yes	11	Symptoms, anxiety/depression, personal/financial problems, time at healthcare appointments
Patient Activation Measure (PAM) (8)	No	22	Managing health condition, active role in health care, ability to manage symptoms, knowledge of prescription medications, knowing when to seek help, communication with healthcare provider, knowledge of health condition, prevention activities, and treatment options, maintaining lifestyle changes
Perceived Kidney/Dialysis Self-Management Scale (PKDSMS) (9)	Yes	8	Difficulty finding effective solutions, self-perceived efficacy, ability to accomplish goals
The Kidney Disease Questionnaire (10)	Yes	26	Measures kidney-specific knowledge about how kidneys work, diet restrictions, transplant, and how to manage dialysis complications
Self-management scale for kidney transplant recipients (11)	Yes	20	Transplant-specific questions relating to self-monitoring and self-care behavior, coping with abnormalities after transplant, stress management

References

1. SMRC. Chronic Disease Self-Efficacy Scale: Stanford Self-Management Resource Centre (SMRC); 1996 [Available from: https://www.selfmanagementresource.com/docs/pdfs/English_-_chronic_disease_self-efficacy_scales_32.pdf.
2. Peng S, He J, Huang J, Tan J, Liu M, Liu X, et al. A chronic kidney disease patient awareness questionnaire: Development and validation. PLoS One. 2019;14(5):e0216391.

Table S1. CKD-related self-management survey instruments

3. Lin CC, Wu CC, Wu LM, Chen HM, Chang SC. Psychometric evaluation of a new instrument to measure disease self-management of the early stage chronic kidney disease patients. J Clin Nurs. 2013;22(7-8):1073-9.
4. Lin CC, Wu CC, Anderson RM, Chang CS, Chang SC, Hwang SJ, Chen HC. The chronic kidney disease self-efficacy (CKD-SE) instrument: development and psychometric evaluation. Nephrol Dial Transplant. 2012 Oct;27(10):3828-34. doi: 10.1093/ndt/gfr788. Epub 2012 Feb 17. PMID: 22344776; PMCID: PMC3808692.
5. Wang SL, Chiu YW, Kung LF, Chen TH, Hsiao SM, Hsiao PN, et al. Patient assessment of chronic kidney disease self-care using the chronic kidney disease self-care scale in Taiwan. Nephrology (Carlton). 2019;24(6):615-21.
6. Herdman M, Gudex C, Lloyd A, Janssen M, Kind P, Parkin D, et al. Development and preliminary testing of the new five-level version of EQ-5D (EQ-5D-5L). Qual Life Res. 2011;20(10):1727-36.
7. Raj R, Ahuja K, Frandsen M, Murtagh FEM, Jose M. Validation of the IPOS-Renal Symptom Survey in Advanced Kidney Disease: A Cross-sectional Study. J Pain Symptom Manage. 2018;56(2):281-7.
8. Hibbard JH, Stockard J, Mahoney ER, Tusler M. Development of the Patient Activation Measure (PAM): conceptualizing and measuring activation in patients and consumers. Health Serv Res. 2004;39(4 Pt 1):1005-26.
9. Wild MG, Wallston KA, Green JA, Beach LB, Umeukeje E, Wright Nunes JA, et al. The Perceived Medical Condition Self-Management Scale can be applied to patients with chronic kidney disease. Kidney Int. 2017;92(4):972-8.
10. Devins GM, Binik YM, Mandin H, Letourneau PK, Hollomby DJ, Barre PE, et al. The Kidney Disease Questionnaire: a test for measuring patient knowledge about end-stage renal disease. J Clin Epidemiol. 1990;43(3):297-307.
11. Kosaka S, Tanaka M, Sakai T, Tomikawa S, Yoshida K, Chikaraishi T, et al. Development of Self-Management Scale for Kidney Transplant Recipients, Including Management of Post-Transplantation Chronic Kidney Disease. ISRN Transplantation. 2013;2013:619754.
