# Supplementary material for: Development and Preliminary Psychometric Testing of an Adult Chronic Kidney Disease Self-Management (CKD-SM) Questionnaire
Source: Can J Kidney Health Dis. 2021 Dec 12;8:20543581211063981. doi: 10.1177/20543581211063981 (PMC8671825; doi:10.1177/20543581211063981)
Supplement: sj-pdf-3-cjk-10.1177_20543581211063981 – Supplemental material for Development and Preliminary Psychometric Testing of an Adult Chronic Kidney Disease Self-Management (CKD-SM) Questionnaire [file sj-pdf-3-cjk-10.1177_20543581211063981.pdf]

Table S3. Pilot study participant feedback on questionnaire content, ease of completion, and suggestions for improvement

Pt ID	Do you think we are missing any important questions about managing CKD? If so, please describe:	Do you think any of the questions are not relevant to managing CKD? If so, please describe:	Did you have trouble completing this questionnaire? If so, please describe:	Do you have any suggestions to improve this questionnaire? For example wording or phrasing changes, or appearance?
101	maybe a quesiton about alcohol, fatty liver disease?	all questions are relevant	I don't know anything about the salt question, I entered "neutral", but maybe it should be "I'm not sure"?	everything is ok
102	"do I thoroughly understand the nature of my CKD?" I don't feel I have chronic disease, so I'm not sure I need to do anything	nothing stands out - all are relevant	no trouble, there is nothing ambiguous about it	thought it was fine, thought it was really good. It is progrerssive, one question leads to the next in terms of relevance.
103			some wording was medical, example glomur…something. May need to change some words	
104			no trouble, it's ok, it makes sense	
105	Do you see a nephrologist. Where I live it is extremely difficult to see one if you are not below stage 3.	No	Very quick and easy	
106				
107	Although this is about the patient,might want to include caregiver and spouse also family members.	All very good questions!	No	It’s all good.
108			No	
109	Am I aware of new drugs or therapies that may be available to me to assist with my kidney health or decline in function	The travel question		
110	There are no questions about mental health, about family and support systems as ckd affects the whole family,		no problems completing it, no questions about demographics or whether the person answering has ckd or esrd, could that skew the results, did I miss that in the introduction perhaps?	Perhaps a brief, one sentence description of the topic it felt odd going from one to the other and having to re-think what I was reading and how to answer
111	My problem is my healthcare provider seems more concerned about other issues vs my kidneys.	No	No	No
112	Do you know who your Neurologist is? Do you know who your primary nurse is. Do you no know who to contact if you are having problems with your access?		No back button to review answers and questions when you get to this section......scroll down fuction on side of this box too sensitive so if yiou have more than two lines it is impossible to review middle line (using mouse is no better)	see previous
113				
114	No	No	No	No
115				
116	No.	no.	no.	no.
117				
118	Think that about covers it. Please do one for transplanted patients.	I think all relavent	Not at all	Nope
119	I think there should be more questions relating to the emotional/mental health aspect of ckd			
120				
121				
122				
123	No but with covid19 some may not be comfortable with telephone clinics	Questions are good	No I have been on dialysis for 6 years and a kidney transplant a year and a half ago	No good survey
124				
125	Mental health			
126				
127	Knowledge about the course of the disease			
128	No	No	No	No
129	Why is everyone afraid to deal with the mental health issues we face?			
130	Ask: do your family members understand what it means to have CKD? Do friends, colleagues?	no	no	no
